# Assessment of prevalence of dental caries and the associated factors among patients attending dental clinic in Debre Tabor general hospital: a hospital-based cross-sectional study

**DOI:** 10.1186/s12903-018-0581-8

**Published:** 2018-07-04

**Authors:** Yilkal Tafere, Selam Chanie, Tigabu Dessie, Haileyesus Gedamu

**Affiliations:** 1 Department of Public health, College of Health Sciences, Debre Tabor University, Debre Tabor, Ethiopia; 2 Department of Nursing, Debre Tabor General hospital, Debre Tabor, Ethiopia; 3 Department of Nursing, College of Health Sciences, Debre Tabor University, Debre Tabor, Ethiopia; 4 Department of Nursing, College of Medicine and Health Sciences, Bahirdar University, Bahirdar, Ethiopia

**Keywords:** Dental caries, Associated factors Debre Tabor, Ethiopia

## Abstract

**Background:**

Dental caries is the most common dental health problem caused by the interaction of bacteria on tooth enamel. Risk factors for dental caries include salivary composition and inadequate fluoride. However, other factors, such as standard of living, behavior, hygiene, eating habits, social status and socio-demographic factors, also contribute to the evolution of caries. Therefore, this study aimed to determine the prevalence of dental caries and associated factors among patients attending the dental clinic in Debre Tabor General Hospital in North West Ethiopia.

**Method:**

An institution based cross-sectional study was conducted among 280 systematically selected patients attending Debre Tabor General Hospital dental clinic from May 8–20, 2017. The data were collected using pre-tested questionnaire and oral examination by a qualified dental professional. Basic hygienic procedures were observed during an oral examination. The teeth were examined for dental caries by the presence of decay, missing and filled teeth. The data were entered into Epi-Info version 3.5 and cleaned and analyzed using SPSS version 20. Descriptive summary of the data and logistic regression were used to identify possible predictors using odds ratio with 95% confidence interval and *P*-value of 0.05.

**Results:**

A total of 280 subjects participated in the study; among whom 129 (46.1%) were female and nearly two-thirds of the respondents 208 (74.3%) attended formal education. The study revealed k8that the overall prevalence of dental caries was 78.2%. Dental caries was lower among respondents who had good oral hygiene status (AOR = 0.05, 95% CI, 0.02, 0.81). Dental caries was higher among participants who earned less than 5000 Eth Birr per month (AOR = 8.43, 95% CI, 2.6, 27.2). Dental caries was lower among respondents who had good knowledge (AOR = 0.51, 95% CI, 0.03, 0.64).

**Conclusions:**

Prevalence of dental caries was high and found public health problem. Socioeconomic status, educational level, and poor oral hygiene practices were associated factors for dental caries. Health promotion about oral hygiene and integration of services are supremely important for the prevention of the problem of dental caries.

## Background

Dental caries is one of the oral health problems which cause the destruction of the hard parts of a tooth by the interaction of bacteria and fermentable carbohydrates [1, 2]. Now a day dental caries on the rise to become major public health problems worldwide, nearly 60–90% of children and about 100% of adults have dental cavities, often leading to pain and discomfort [3].

The problem related with dental caries leads to a decrease in the quality of life of the affected individuals and high economic costs for equally individuals and society, with disparities related to well-known issues of socioeconomics, immigration, lack of preventive efforts, and dietary changes [4]. The burden of dental caries in children is incredibly high. The Pain from dental caries can affect school attendance, eating and speaking, and, then impair growth and development [5, 6].

Even though the overall prevalence of dental caries decrease in developed countries, caries continues to be an important public health problem in most developing countries [7]. A study conducted in Lithuania showed that the overall prevalence of dental caries was 78.3%. [8]. Another study conducted in Brazil among adults aged 35 to 44 years showed that 82.0% consumed sugary foods up to four times a day. A study done in Brazil showed that 75% of the participants had enamel defects [9, 10]. Another study was done in Bulgaria also showed that Age, sex, and education were associated with tooth decay. Higher education was associated with a lower chance of having “missing” teeth. More frequent tooth brushing was associated with a lower chance of having decayed [11].

The prevalence of dental caries in Sudan was reported as 30.5% [12]. Another study done in Kenya revealed that the prevalence of dental caries was 37%. The main reason attributed was lack of knowledge on the causes and preventive methods of the disease [13].

An increasing utilization of sweet foods in the developing countries, poor tooth brushing habits, poor oral hygiene and low level of awareness are some of the factors that increased the levels of dental caries. In addition to this the way of life, eating habits, social status and socio-demographic factors also contribute to the development of caries. Caries can be prevented by decreasing sugar intake and brushing teeth after every meal using the appropriate techniques and regular check-ups [5–9, 14].

Although the trend is not clear in developing countries such as Ethiopia, Oral diseases have a growing impact on the health and well-being of people in the region and in particular on vulnerable and marginalized groups of the population. The burden of dental caries has been increasing due to the unlimited utilization of sugary foods, poor oral care practices and inadequate health service utilization [15, 16]. A study done in Finote Selam, Ethiopia showed that 48.5% of the students had dental caries. The prevalence was higher in female 54.6%. Lack of tooth brushing habit (AOR = 3.5, 95% CI: 1.9–6.4), frequent consumption of sugared foods (AOR = 3.4, 95% CI: 1.3–5.6) and residency (AOR = 1.8, 95% CI: 1.0–3.3) were found to have a significant association with dental caries [17]. Another study done Bahirdar city showed that the prevalence of dental caries was 21.8%, Poor habit of tooth cleaning was significantly associated with dental caries [18]. The study done in Gondar showed that there was a statistically significant association between dental caries and educational status (AOR = 0.37, 95% CI, 0.17, 0.80). Dental caries among children whose father were above grade 12 were 63% times at a lower risk compared to illiterates [19].

Untreated dental caries might lead to dental pain, which in turn results in impacts of affected play and sleep, avoidance of certain types of food and decreased performance [15]. In Ethiopia, existing dental health services are limited. Even though dental caries is high in the country, much is not known about the factors affecting it in the study area. Therefore this study was aimed to assess the prevalence of dental caries and its associated factors among patients attending Dental Clinic in Debre Tabor General Hospital.

## Methods

### Setting

This institution based cross-sectional study was conducted in Debre Tabor General Hospital Dental Clinic from May 8^th^ and 20th 2017. Debre Tabor General Hospital that provides health service to over 1 million people and is located 654 Kms northwest of the capital city, Addis Ababa. Debre Tabor General Hospital is found in Debre Tabor town which is the capital of the South Gondar Zone. The hospital provides different inpatient and outpatient services including dental health services to the population in the surrounding area of Debre Tabor town and the nearby districts.

### Participants

#### Source population

The source population was all patients who attending Debre Tabor Hospital, Dental clinic.

#### Study population

Systematically selected patients age 18 and above years old getting services from Debre Tabor hospital, dental clinic.

#### Exclusion criteria

Patients who were unable to communicate were excluded.

### Sampling technique and procedure

A sample size of 288 was determined using single population proportion formula.

$$ \mathrm{n}=\frac{{\mathrm{Z}}^2\mathrm{p}\left(\mathrm{p}-1\right)}{{\mathrm{d}}^2} $$

with the following assumptions: proportion (P) The prevalence of dental caries to be 21.8% as estimated from the study done in Bahir Dar, Ethiopia [19], a confidence level (CI) of 95%, and marginal error (d), 5 and 10% non-response rate.

Debre Tabor General Hospital was selected purposively. Daily patient flow to Debre Tabor general hospital dental clinic was calculated and. Finally, a systematic selection was done to identify study subjects.

### Variables

The dependent variable was the prevalence of dental caries, and the independent variables were demographic, socioeconomic characteristics, information on dental caries, hygienic practice, and feeding habit and associated factors.

### Operational definitions

#### Dental caries

The presence of tooth decay, missing and filled teeth at the time of the oral examination.

#### Good oral hygiene status

If no food particles and no accumulation of dental plaque and/or calculus is visible on the tooth surfaces at the time of the oral examination.

#### Poor oral hygiene status

If the presence of food particles in the mouth and there is a visible accumulation of dental plaque and/or calculus on the tooth surfaces at the time of oral examination.

#### Good knowledge

Those respondents who got a score greater than the mean (greater than five) from ten “yes/no” knowledge related questions about dental caries.

#### Poor knowledge

Those respondents who got a score less than or equal to the mean (got less or equal to five) from ten “yes/no” knowledge related questions about dental caries.

### Data collection

Oral examination was done by a doctor of dental medicine. Hygienic statuses of the respondents were watched during an oral examination. A structured, pre-tested Amharic version questionnaire which was first drafted in English was used to collect data. The questionnaire was pre-tested and appropriate corrections were made for the main study. Data collectors were recruited in consultation with their immediate supervisors by considering their ability in establishing a good relationship with their clients, and their ability to record responses on questionnaire accurately. They were also trained by the principal investigators for two days on how to interview, handling, maintaining confidentiality and ethical issues. A day to day on site supervision was made during data collection by the investigators and one dentist professional.

### Data analysis

Data were checked and entered using EPI-Info version 3.5.3 statistical software. Data were cleaned and edited accordingly and exported to SPSS version 20 for analysis. Descriptive analysis such as numerical summary measures, frequencies and proportions were computed. The association between the independent and outcome variables was first investigated using bivariate analysis. Those variables with *p* value ≤ 0.25 were included into multivariable analysis to determine the predictor variables for the outcome variables. Finally further analyses were carried out using multivariable analysis at significance level of *p*-value ≤ 0.05.

### Ethical consideration

Ethical approval for this study was obtained from research evaluation and an ethical review committee of Debre Tabor University College of Health Sciences. Permission was obtained from Debre Tabor general Hospital. Verbal informed consent was obtained from each participant after providing complete information about the purpose and procedures of the study. At the end of the data collection session, all study participants were advised on how they can maintain their oral hygiene. The consent procedure was approved by the research evaluation and ethical review committee.

## Results

### Socio-demographic characteristics

A total of 280 subjects participated in the study; among whom129 (46.1%) were female andnearly two-thirds of the respondents 208 (74.3%) attended formal education. The mean age of the respondent was 33.23 with ±12.5 standard deviations (SD) and 103 (36.8%) of the respondents were in the age group of 20 to 29 years. Two hundred sixty-three (93.9%) were Orthodox Christian in religion, 271 (96.8%) were Amhara in ethnicity. One hundred ninety-three (69%) of the respondents were currently married. One hundred eighty-two (65.0%) of the respondents had a monthly income of less than 5000 Ethiopian birrs (Table 1).

**Table 1:** Socio-demographic characteristics of patients attending the dental clinic in DebreTabor General Hospital, Northwest Ethiopian, 2017

Variables	Frequency	Percent (%)
Age		
< 20	35	12.6
20–29	103	36.8
30–39	76	27
40–49	31	11
50–59	20	7.2
≥ 60	15	5.4
Sex		
Female	129	46.1
Male	151	53.9
Residence		
Urban	171	61.1
Rural	109	38.9
Religion		
Orthodox	263	93.9
Muslim	11	3.9
Protestant	6	2.2
Marital status		
Married	193	69
Single	76	27.1
Divorced	7	2.5
Widowed	4	1.4
Educational status		
Not attend formal education	72	9.7
Attend formal education	208	74.3
Occupation		
Farmer	96	34.3
Government employed	63	22.5
Merchant	80	28.6
Student	30	10.7
Other	11	3.9
Ethnicity		
Amhara	271	96.8
Tigray	6	2.2
Others	3	1
Monthly income in Eth.Birr		
< 5000	182	65.0
> 5000	98	35.0

### Prevalence of dental caries, food consumption and practices related to oral hygiene

The results of this study revealed that the overall prevalence of dental caries was 219 (78.2%) among this the majority 80(36.6%) of the teeth affected by dental caries was molar. One hundred twelve (40%) of the study subjects had good knowledge about causes and prevention of dental caries. Seventy (24.9%) of the respondents had tooth brushing of which 20(28.6) of the respondents brush their teeth once per day (Table 2).

**Table 2:** Prevalence of dental caries, food consumption and practices related to oral hygiene among patients attending the dental clinic in Debre Tabor General Hospital, Northwest Ethiopian, 2017

Variables	Frequency	Percent (%)
Knowledge about dental caries		
Good	104	38.2
Poor	176	62.8
Oral Hygiene status		
Good	112	40
Poor	168	60
Tooth brushing habit?		
Yes	70	24.9
No	210	75.1
The frequency of tooth brushing (*n* = 70)		
Once per day	20	28.6
Sometimes	50	71.4
Time of tooth brushing (n = 70)		
Morning	59	84.3
Mixed	5	7.1
Not fixed	6	8.6
Alcohol frequent consumption		
Yes	206	73.6
No	74	26.4
Sugared food consumption		
Yes	168	60
No	112	40
Frequency of consumption sugared foods (*n* = 168)		
Once per day	76	45.2
Twice per day	33	19.6
Sometimes	59	35.2
The family history of Dental Disease		
Yes	27	9.6
No	253	90.4
Dental caries (any type)		
Yes	219	78.2
No	61	21.8
Type of tooth decayed /missed (*n* = 219)		
Incisors	39	17.9
Canines	30	13.5
Premolars	70	32
Molar	80	36.6

### Logistic regression analysis of factors associated with dental caries

The factors significantly associated with dental caries in bivariate analysis were entered into a multivariable logistic regression model as independent variables for the outcome of dental caries.

This finding demonstrated a significant association between respondent’s level of education and dental caries (AOR = 0.24, 95% CI, 0.12, 0.49). Dental caries among respondents who had attended any formal education was 76% times lower risk of developing dental caries compared to those who were not attended formal education. Dental caries was lower among respondents who had good oral hygiene status as compared to those whose oral hygiene status was poor (AOR = 0.05, 95% CI, 0.02, 0.81).

A patient who lives in urban had 1.6 times (AOR = 1.6(1.2, 4.3)) chance of developing dental caries than those patients who were living in rural. Dental caries was higher among respondents who earned less than 5000 Eth Birr per month as compared to those earning greater than 5000 Eth Birr per month (AOR = 8.43, 95% CI, 2.6, 27.2). Dental caries was lower among respondents who had good knowledge about dental caries as compared to those patients who had poor knowledge about dental caries (AOR = 0.051, 95% CI, 0.03, 0.64) (Table 3).

**Table 3:** Bivariate and multivariate analyses of variables associated with dental caries among patients attending Debre Tabor General Hospital dental clinics, Debre Tabor, North West Ethiopia, 2017

Variables	Dental caries		COR (95%)	AOR (95%)	*p*-value
Monthly income	Yes	No			
< 5000 Eth birr	155	27	3.05, (CI, 1.7,5.5)	8.43(CI,2.61,27.20)	0.000*
> 5000 Eth birr	64	34	1.00	1.00	
Educational status					
Not Attend formal education	41	31	0.22(CI, 0.12,0.41)	0.24(CI,0.12,0.49)	
Attend formal education	178	30	1.00	1.00	0.000*
Resident					
Urban	141	30	1.95(CI 1.6,4.6)	1.6(CI,1.2,4.3)	0.01*
Rural	78	31	1.00	1.00	
Sex					
Female	108	21	3.98 (CI 1.85,6.73)	3.2 (CI,2.33,4.54)	0.001
Male	111	86	1.00	1.00	
Oral hygiene status					
Good	59	53	0.06(CI0.01,0.96)	0.05(CI,0.02,0.81)	0.001*
Poor	160	8	1.00	1.00	.
Knowledge about dental caries					
Good	86	18	0.7,(CI 0.40,.09)	0.51(CI,0.03,0.64)	0.001*
Poor	133	43	1.00	1.00	

*It means siginificantly associated

## Discussion

This study attempted to assess the prevalence and associated factors of dental caries amongpatients attending Debre Tabor General Hospital dental clinic. The overall prevalence of dental caries found in this study was 78.2%, which was consistent with study in Lithuania (78.3%), [8] and higher than the studies, (75%) in Brazil [9], 37% in Kenya, 30.5% in Sudan [12, 13], (48.5%) in Finote Selam, [17], Ethiopia, and 21.8% in Bahirdar city Ethiopia [18]. The high prevalence of dental caries in this study might be due to the fact that there were variations in study population, time and study setting, in this study since it is institutional based there might be high patient flow in health institutions compared to the community level; this indicates that there is a need to promote oral health. The difference with the Brazil, Kenya, and Sudan studies might be due study population variation and, the socio-demographic differences between those countries.

In the study, factors associated with dental caries were knowledge about prevention and causes of dental caries, oral hygiene status, monthly income, place of resident, educational status, and marital status.

The study found that dental caries was lower among respondents who had good oral hygiene status were 95% times less likely to be affected by dental caries as compared to those patients whose oral hygiene status was poor (AOR = 0.05, 95% CI, 0.02, 0.81). This finding is typically similar to the studies done in Finote Selam and Bahirdar [17, 18]. The study revealed that patients who did not attend any formal education were 76% times at a higher risk compared to those who attended formal education (AOR = 0.24, 95% CI, 0.12, 0.49) which is consistent with findings from a study conducted in Gondar [19].

This study showed that Patient who lives in urban had 1.6 times (AOR = 1.6**,** 95% CI 1.2, 4.3) chance of developing dental caries than those patients who were living in rural. This could be that patients who live in urban settings could have access to consume commonly more sugary foods that cause dental caries.

There was also a significant difference between the monthly income of the households and dental caries**.** Dental caries was higher among respondents who earned less than 5000 Eth Birr per month as compared to those earning greater than 5000 Eth Birr per month (AOR = 8.43, 95% CI, 2.6, 27.2). This result is in line with the study done in Gondar town [19]. As the income of the families increasing, people are less likely to be affected by dental decays. This could be elaborated that those who have better monthly income can have potential to buy tooth cleaning materials. Dental caries was lower among respondents who had good knowledge about dental caries as compared to those patients who had poor knowledge about dental caries (AOR = 0.051, 95% CI, 0.03, 0.64). Having good knowledge about dental caries could help to have better health care seeking behavior of the community.

However, this study does have some inherent limitations. First, we did not use any of the recognized Oral Hygiene Index to assess oral hygiene status. Finally, though there are wide ranges of factors which affect the prevalence of dental caries among patients attending a dental clinic, only individual-level factors were addressed in this study. Hence, taking into consideration factors from the service providers’ side and structural barriers would have been important.

## Conclusions

In conclusion, this study showed, the prevalence of dental caries was high and found public, knowledge about dental caries, educational status, oral hygiene status, place of residence, and monthly income were important predictors of the prevalence of dental caries among patients attending Debre Tabor General Hospital Dental Clinic. Therefore, integrating oral health promotion service with other health services at the grass root levels could have a significant impact and are likely to benefit community’s oral health problem at large. Health promotion about oral hygiene is supremely important for the prevention of the problem of dental caries.

## Abbreviations

DMFT: Decayed, Missing, and Filled Teeth
OR: Odds ratios
SD: Standard deviation
SPSS: Statistical package for social sciences
WHO: World Health Organization
